# How Can We Identify and Communicate the Ecological Value of Deep-Sea Ecosystem Services?

**DOI:** 10.1371/journal.pone.0100646

**Published:** 2014-07-23

**Authors:** Niels Jobstvogt, Michael Townsend, Ursula Witte, Nick Hanley

**Affiliations:** 1 Oceanlab, University of Aberdeen, Aberdeen, United Kingdom; 2 Aberdeen Centre for Environmental Sustainability (ACES), University of Aberdeen, Aberdeen, United Kingdom; 3 National Institute of Water and Atmospheric Research (NIWA), Hamilton, New Zealand; 4 Department of Geography and Sustainable Development, University of St Andrews, St Andrews, United Kingdom; Northwest Fisheries Science Center, NOAA Fisheries, United States of America

## Abstract

Submarine canyons are considered biodiversity hotspots which have been identified for their important roles in connecting the deep sea with shallower waters. To date, a huge gap exists between the high importance that scientists associate with deep-sea ecosystem services and the communication of this knowledge to decision makers and to the wider public, who remain largely ignorant of the importance of these services. The connectivity and complexity of marine ecosystems makes knowledge transfer very challenging, and new communication tools are necessary to increase understanding of ecological values beyond the science community. We show how the Ecosystem Principles Approach, a method that explains the importance of ocean processes via easily understandable ecological principles, might overcome this challenge for deep-sea ecosystem services. Scientists were asked to help develop a list of clear and concise ecosystem principles for the functioning of submarine canyons through a Delphi process to facilitate future transfers of ecological knowledge. These ecosystem principles describe ecosystem processes, link such processes to ecosystem services, and provide spatial and temporal information on the connectivity between deep and shallow waters. They also elucidate unique characteristics of submarine canyons. Our Ecosystem Principles Approach was successful in integrating ecological information into the ecosystem services assessment process. It therefore has a high potential to be the next step towards a wider implementation of ecological values in marine planning. We believe that successful communication of ecological knowledge is the key to a wider public support for ocean conservation, and that this endeavour has to be driven by scientists in their own interest as major deep-sea stakeholders.

## Introduction

The concept of ecosystem services (ES) has inspired a movement away from conservation for the sake of nature’s inherent value to one that explicitly identifies, links and communicates the benefits of conservation to human wellbeing [1]–[3]. The endeavour of describing, quantifying and valuing the economic benefits that nature provides to society through ES has been identified as a powerful tool to make ecosystems count in cost-benefit analysis for environmental decision making [1], [4]. Throughout this paper, however, the term ‘value’ is used in a broader sense, as a holistic concept which can include social, ecological and economic values. This broadening of the concept of value is needed because for the remotest places on earth like the deep sea, it is particularly challenging to make direct links between changes in system functioning and effects on the delivery of final ES (and thus on human well-being) [5], [6].

The deep sea accounts for nearly 91% of the world’s oceans with depths ranging from 200 m to almost 11,000 m. Despite its remoteness and size, its ecosystems are far from being unaffected by anthropogenic impacts such as fishing, climate change, and pollution [7]–[10]. To date many knowledge gaps remain around the functioning of deep-sea ecosystems. This is partially explained through the high costs, difficulties, and risks that are associated with deep-sea research. This lack of ecological knowledge means that we also know very little about the social and economic value of protecting the deep sea. By identifying and quantifying the ES benefits provided by the deep sea it is likely that appreciation for these benefits will change. This should lead to a larger emphasis on mitigating anthropogenic impacts in the oceans.

**Figure 1:**
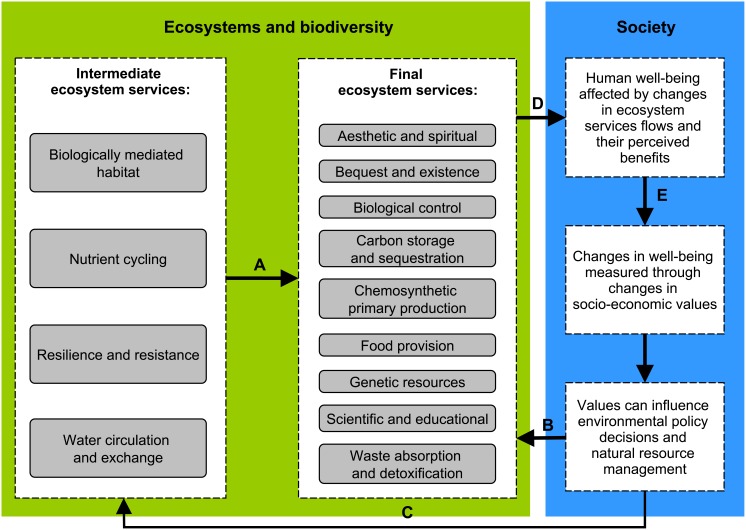
The ecosystem services framework for the example of deep-sea ecosystem services (ES). Environmental policies can either influence the management of final ES directly (arrow B) or indirectly via the intermediate ES (arrow C). The latter requires a sufficient understanding of the dependencies between intermediate and final ES (arrow A). Our understanding for the benefits provided by deep-sea ES (arrow D) and the values associated with them (arrow E) is currently very limited. The framework was simplified from [53] and adapted to the ES used for this expert consultation.

The major challenges of accounting for deep-sea ES stem from most people’s lack of awareness about the deep-sea environment, and from the prevalence of intermediate services relative to easier-to-appreciate final services. Intermediate services in this paper refer to the indirect services that the ecosystem provides, such as habitat provision and nutrient cycling (the Millennium Ecosystem Assessment [1] refers to this category as supporting services). Intermediate services are the functional basis of the final services supplied by the system (Figure 1). The final services are considered as the ecosystem’s contribution to human well-being [11] and include the ES categories of provisioning (e.g. commercial fish species), regulating (e.g. waste absorption and detoxification) and cultural (e.g. aesthetic values) [1]. There is a need to improve the integration of intermediate services and the processes that sustain them into the way in which we assess ES [12], since this ecological understanding is essential for demonstrating how human well-being ultimately depends on ecological processes and biodiversity [13], [14].

In this paper we analyse the links between ES and the underlying processes that sustain them. This analysis was undertaken with the help of an expert panel of deep-sea scientists, focussing on submarine canyons. Within the deep sea, submarine canyons are an important ecosystem, which host unique reservoirs of biodiversity [15]–[18]. Along with much of the deep sea, they remain mostly underexplored and unprotected [9], [19], [20]. We selected this deep-sea ecosystem to test the Ecosystem Principles Approach (EPA) [21], [22]. The EPA has recently been developed as a way of incorporating and translating ecological knowledge into easily understandable ‘units’ of information (‘ecosystem principles’) suitable for a wide range of audiences and thus for use in an ecosystem management context. The focus is on known and broadly-accepted information, with scientists from a wide field of expertise condensing this knowledge into principles that explain the linkages between ES, and their dependencies on underlying processes. The ecosystem principles also provide marine managers with qualitative information on temporal, spatial, and causal dependencies of ES flows. In the New Zealand case study by Townsend and colleagues [22], the EPA highlighted the importance of accounting for intermediate services in marine management, which were often provided by different geographical areas relative to the location at which final services were taken into account in the ES assessment. Economic theory suggests that intermediate ES can and should be valued through the final services that they support and the resultant direct benefits to people [40]. Indeed, ES are often perceived as a purely economic concept [23], but they also have social and ecological values, which when integrated with economic values produce a more holistic ecosystem assessment that can better inform natural resource management decisions [24]. This dominance of economic approaches and monetary valuation of ES stems from the often-felt pressure among the nature conservation sector to “speak the same language” as business and policy sectors in order to make conservation count [25]–[29].

In contrast, the EPA’s advantage lies where economic valuation reaches its limits, by offering a more holistic picture which might help non-experts to better understand the high ecological value that scientists associate with the deep sea. Links between ecosystem processes and ES are less well known for the deep sea. For this reason it may be more helpful to highlight the links between deep-sea ES and processes themselves, rather than presenting decision makers with a set of economic values that are likely to underestimate the ecosystems’ holistic value due to the omission of important ecological aspects of the deep sea [23]. In this paper we test the applicability of the EPA to little known and remote deep-sea ecosystems, such as submarine canyons, and demonstrate how the approach can provide decision makers with an accessible knowledge base for conservation decisions, despite some deficiencies in scientific data and associated uncertainty. We further discuss the approach’s utility for expert consultation and cross-disciplinary knowledge transfer.

## Methodology

### 2.1 Case study area: The Nazaré Canyon

**Figure 2:**
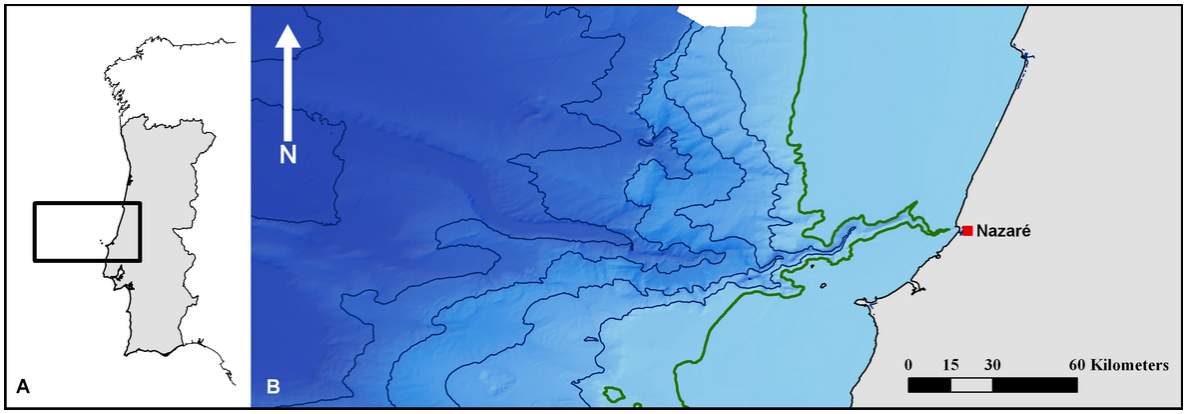
Nazaré Canyon. (**A**) Overview map of Portugal and the Nazaré Canyon area. (**B**) Nazaré Canyon bathymetry map with the Portuguese coastline to the east. Contour lines (blue) at 1000****m intervals; the 200****m depth contour, indicating the shelf edge, is marked in green. Data courtesy of Instituto Hidrografico, Lisbon and National Oceanography Centre, Southampton.

The Nazaré Canyon on the Portuguese continental margin (Figure 2), also described as “Europe’s Grand Canyon” [16], was chosen as the case study area of this paper to test the applicability of the EPA for deep-sea ecosystems. The Nazaré Canyon has attracted scientific interest due to its habitat heterogeneity and is considered to be a biodiversity hotspot [16]. Like other submarine canyon ecosystems it plays an important role in transportation processes at the continental margins [30], [31]. The canyon is shallowest at 1 km off the Portuguese coast (50 m depth) and with a total length of 210 km it extends into the Iberian abyssal plain where it reaches depths of over 4,900 m [32].

#### 2.2.1 Sampling and survey structure

**Figure 3:**
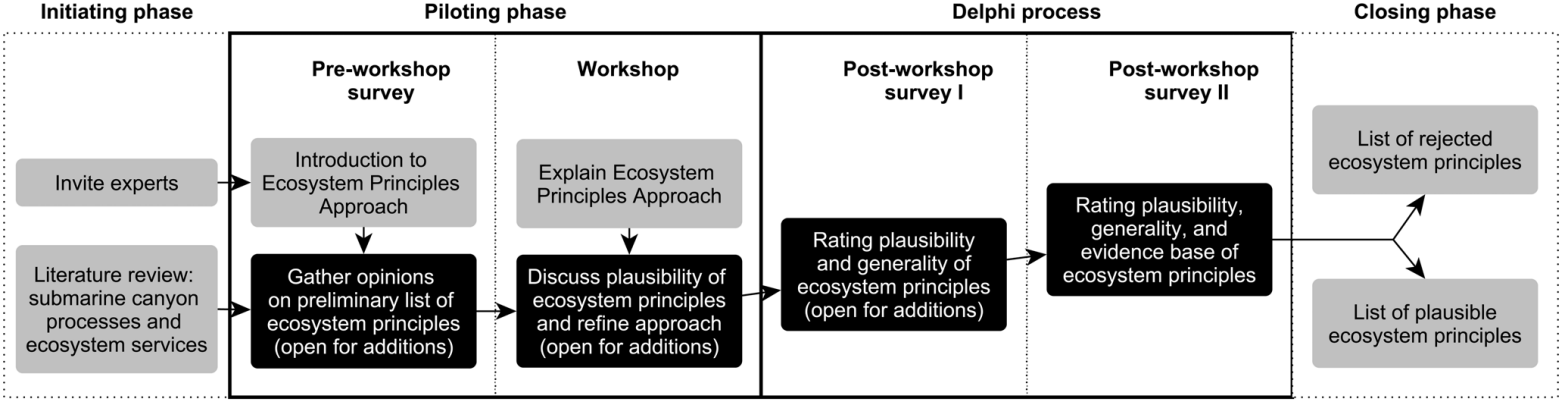
The survey phases of the submarine canyon expert consultation. Survey steps where experts were directly involved are highlighted as black boxes.

Twenty-three researchers with knowledge of the Nazaré Canyon, covering a broad range of disciplines such as ecology, biology, microbiology, biogeochemistry, geography, geomorphology, geology, sedimentology and oceanography, were invited to participate in a HERMIONE (Hotspot Ecosystem Research and Man’s Impact On European Seas project; URL: www.eu-hermione.net; last access July 2013) workshop in September 2012 and in an online pre-workshop survey (Figure 3). These opportunities were used to gather ideas and feedback for the two main surveys that followed. We used an email-based Delphi process to gather structured information by consensus from the invited expert panel in two consecutive rounds of surveying (post-workshop survey I and II; Figure 3). The Delphi process was originally developed as an interactive forecasting technique, where an expert panel goes through iterative survey rounds. The group results of each round are fed back to participants who are able to adapt their responses in the next survey round. The main idea of the Delphi process is to lead the group towards a consensus through the indirect exchange of information via a process coordinator. This process allowed us to subsequently include experts’ requests for changes and additional information into the post-workshop surveys (Figure 3). The Delphi process makes it less likely that some researchers dominate the discussion and the outcomes, by maintaining anonymity throughout the communication process, thus avoiding the potential peer pressure of an expert workshop setting (further detail on the Delphi process in [33]). The survey questionnaires are available on request from the corresponding author.

#### 2.2.2 Ethics statement

The nature of this research did not require ethical approval according to the University of Aberdeen Research Ethics Framework (*University of Aberdeen Research Ethical Review Checklist, Appendix A,* pp. 26–28; URL: www.abdn.ac.uk/documents/research-governance-framework-appendix4.pdf; last access March 2013). All study participants were recruited using an opt-in strategy and therefore consent was not explicitly recorded. Workshop and surveys did not include any sensitive personal questions. We asked participants to state their age, years of research experience, and field of studies; answering these questions was optional. Participants were provided with information on study objectives, sponsors, the participants’ role, survey and workshop durations, potential benefits to the participants, summarised methodology, destination of gathered data and research results, the potential science impact and a contact address for further questions. Throughout the post-workshop surveys participants were identifiable via their email addresses. However, data on stated opinions and personal information was stored anonymously and kept confidential at all times. We chose email as the preferred communication method to facilitate the exchange of information during the Delphi phase of the expert consultation.

### 2.3 Submarine canyon ecosystem services

The experts helped to identify ES that were either perceived as less important or not relevant for the submarine canyon based on Table 1. Subsequently, the ES ‘genetic resources’, ‘biological control’, ‘aesthetic and spiritual’, ‘scientific and educational’, and ‘chemosynthetic primary production’ were excluded from Table 1 as less important relative to the other deep-sea ES. Accordingly eight ES (Table 1) were taken forward as a focus for the development of ecosystem principles.

**Table 1:** Submarine canyon ecosystem services.

Ecosystem services	Descriptions
**Provisioning services:**	
Carbon sequestration and storage	The uptake, storage, and burial of organic material within the canyon.
Food provision	The provision of marine organisms for human consumption.
Genetic resources and chemical compounds*	The use of canyon organisms in biotechnological, pharmaceutical, or industrial applications.
**Regulating services:**	
Biological control*	The control of diseases and invasive species.
Waste absorption and detoxification	The burial, decomposition and transformation of waste within the canyon ecosystem.
**Cultural services:**	
Aesthetic and spiritual*	The canyon ecosystem aesthetic and spiritual or inspirational source for religion, arts, movies, documentaries, books and folklore.
Bequest and existence	Safeguarding the canyon ecosystem for future generations and for the existence of marine species.
Scientific and educational*	The cognitive use of the canyon ecosystem for science and education.
**Intermediate services:**	
Biologically mediated habitat	Canyon habitats formed by marine organisms that provide nursery and refuge sites for other marine life.
Nutrient cycling	The storage and recycling of nutrients by canyon organisms.
Chemosynthetic primary production*	Primary productivity that is not dependent on energy from the sun.
Resilience and resistance	The amount of disturbance that the canyon ecosystem can cope with and its ability to regenerate after disturbance.
Water circulation and exchange	The currents, such as up-and down-welling, dense shelf water cascading, and mixing of water masses.

Services are grouped into four categories: provisioning, regulating, cultural and intermediate.

Listed items taken from [5], [7], [36], [52] with alterations.

*Deep-sea ES that were not taken forward for the development of submarine canyon principles.

### 2.4 Ecosystem Principles Approach

One of the main goals of the expert consultation was to develop a list of submarine canyon ecosystem principles, which could then be linked to ES. As an initial step, a review on the submarine canyon literature identified relatively well-explored ecosystem processes and relationships. The review findings were then discussed in the expert workshop and principles added or refined according to experts’ suggestions. The following paragraph shows how the concept of ecosystem principles was described to canyon experts: “[An ecosystem principle] explicitly defines a key element of how we expect the ecological system to operate” [22]. The workshop invitation summarised the EPA and included the methodology paper by Townsend and colleagues [22] as preparation for the workshop. During the workshop we provided further information in the form of a presentation that helped to increase participants’ familiarity with the EPA. Principles were excluded from the initial list after each of the two consecutive post-workshop surveys (Delphi process; Figure 3) when fewer than 50% of the experts agreed with the plausibility of the principle. Experts were also able to propose new principles or suggest changes to the list of principles that was identified from the literature and subsequently refined throughout the piloting and Delphi phases (Figure 3).

We also asked the expert panel to categorise ecosystem principles according to their level of generality. The following categories were available: (i) general deep-sea principle, (ii) general canyon principle, (iii) shelf-incising canyon specific principles, to (iv) Nazaré Canyon-specific principle. The option with the highest frequency was then presented as the group vote in the subsequent survey.

Only in the second stage of the Delphi process were experts asked to distinguish their rating based on evidence on the one side and their expert view (as individuals) on the other side. The evidence base was rated on a five-point scale from ‘very poor’ to ‘very good’. For the presentation of the group result, this was then divided into three categories of good, intermediate or poor evidence, according to the average group scores. These evidence scores had no influence on the decision to include or exclude any principle, but were introduced to separate personal opinion from the levels of evidence that existed in support of the principle.

During the workshop experts stressed the importance of the connectivity function of submarine canyons at the continental margin and we therefore chose ‘water circulation and exchange’ as an example to demonstrate how ecosystem principles link the supply of ES with their underlying processes. In the final results section, those ecosystem principles which in their description indicated a relationship between the ES ‘water circulation and exchange’ and other services were linked. The ecosystem principles relevant to this particular ES were assigned by the authors of this paper.

## Results

### 3.1 Sample characteristics

The workshop was attended by 14 deep-sea researchers, 11 of whom had completed the pre-workshop pilot survey (Figure 3). All 14 workshop attendees were invited to participate in the two stages of the Delphi process (Figure 3), which 11 did for the first round and 10 for the second round. The average survey participant had 21 years of research experience and the survey covered academics from senior professors to PhD researchers, with male and female researchers equally represented.

### 3.2 Ecosystem principles

Over the course of the Delphi process, 21 ecosystem principles were identified from the literature and were then assessed and refined by the expert group (Tables 2 and 3). To highlight the nature of ecosystem principles, we present principle P1 as an example, which was rated to be plausible by all experts: ‘canyons host a large number of different habitats and as a result increase species diversity at a regional scale’ (for further principle descriptions we refer the reader to Tables 2 and 3). Four principles were discarded, whereas 17 principles were rated as plausible. Ten ecosystem principles fell into the category ‘general submarine canyon principles’, five into category ‘general deep-sea principles’, and two into ‘shelf-incising canyon specific principles’ (Tables 2 and 3). This indicated that the majority of ecosystem principles were at an appropriate level to describe processes and linkages between ES for submarine canyons in general and that they can be readily transferred to other canyons. The Delphi process had the expected effect of driving opinions closer towards consensus. Seven of the ten experts who participated in Delphi-rounds I and II (Figure 3) were closer to the group rating after the second Delphi-round.

**Table 2:** Submarine canyon ecosystem principles with expert ratings on their plausibility and evidence base.

ID	Ecosystemprinciples	Plausibility	Evidence(mean score ± SE)
	**General submarine canyon principles:**		
**P1**	Canyons host a large number of differenthabitats and as a result increase speciesdiversity at a regional scale.	100%	GOOD (3.8±0.2)
**P2**	The canyon topography tends to have afocusing or channelling effect forsediment and organic material.	100%	GOOD (3.8±0.2)
**P3**	The strength of large scale transportationevents varies and occurrence ranges froma yearly to decadal pattern. They can betriggered by storms, high sediment load inthe water column, cooling and increasingsalinity of surface waters, or slope failures.	100%	GOOD (3.9±0.3)
**P4**	The transport of organic material from shallowerwaters to the deep seabed, which is mainlydriven by large scale transportation events,is an important source of food fordeep-sea organisms.	100%	MEDIUM (3.3±0.3)
**P5**	Canyons can serve as fish feeding ground,refuge and nursery area and thereforeoften show higher abundanceof fish than their surroundings.	90%	MEDIUM (2.8±0.3)
**P6**	Canyons can enhance the mixingof water masses and as a result influencethe exchange of nutrients, heat andsalt between the shelf and the deep sea.	90%	MEDIUM (3.4±0.4)
**P7**	The canyon topography affects up- anddown-welling of water masses at thecontinental margin. Upwelling eventsaround the canyon head enhancesproductivity locally; as a result fishabundance can be higher.	90%	MEDIUM (3.3±0.3)
**P8**	By transporting large amounts oforganic material from the shelf intodeeper waters, canyons act as temporarystores of sediment and carbon. It can takedecades or even centuries until thetransported material reaches the abyssalplain, where it is then depositedon geological time scales.	80%	GOOD (3.8±0.2)
**P9**	Food quantity and quality tends to behigher within some canyon areascompared to the surrounding slope.This can enhance the biomass ofthe benthic and pelagic fauna.	80%	MEDIUM (3.3±0.4)
**P10**	Many species that are found incanyons are not found on the slope.They are therefore contributing toregional diversity.	80%	MEDIUM (3.1±0.4)

ID = principle identification number. The plausibility rating: ten experts participated in the full rating process (i.e. 100% = 10 experts). The evidence rating (1–5 from ‘very good’ to ‘very poor’): poor (mean score <2.5), medium (2.5≤ mean score <3.5) and good (mean score ≥3.5); SE = standard error.

**Table 3:** Submarine canyon ecosystem principles continued from Table 2.

ID	Ecosystemprinciples	Plausibility	Evidence(mean score ± SE)
	**Shelf-incising canyon specific principles:**		
**P11**	Canyons function as major transportpathways between the shelf and the deep sea.	100%	GOOD (4.0±0.3)
**P12**	Sediment, organic material, and pollutantsthat are transported alongshore gettrapped by the canyon and transporteddown the canyon slope.	90%	MEDIUM (3.4±0.3)
	**General deep-sea principles:**		
**P13**	Areas with reef forming or habitatcreating organisms can supporthigher diversity than their surroundings.These habitats are most common onhard substrates, such as areas withsteep slopes, rocks, boulders,vertical walls, or overhangs.	100%	GOOD (4.1±0.3)
**P14**	The biomass of invertebrates livingin and on the seafloor can constitutean important food source forcommercially important deep-sea species.	100%	GOOD (3.7±0.3)
**P15**	The organisms inhabiting soft substratesplay a major role for the recyclingof nutrients. The process is largelydominated by bacteria, but is toa smaller extent also attributed tothe animals living in and on the sediment.	70%	GOOD (3.5±0.3)
**P16**	Higher biodiversity can support higherrates of ecosystem processes.	70%	POOR (2.4±0.5)
**P17**	Higher biodiversity increases theinsurance value of an ecosystemby increasing the likelihood thatthe ecosystem is able to provide thesame ecosystem functionsafter an ecosystem impact occurred.	60%	POOR (2.0±0.4)
	**Rejected ecosystem principles** * **:**		
**P18**	Diversity tends to be lower inareas with high food availability.	10%	Not assessed
**P19**	Space and resource occupancy bynative species can decrease invasion risk.	30%	POOR (1.4±0.2)
**P20**	Where strong bottom currents arecommon, food availability andsubstrate characteristics becomeless important and current speedbecomes the main driver forspecies abundance and diversity.	40%	Not assessed
**P21**	The disturbances caused by strongbottom currents keep speciesdiversity and abundance at low levels.	40%	POOR (2.3±0.4)

ID = principle identification number. The plausibility rating: ten experts participated in the full rating process (i.e. 100% = 10 experts). The evidence rating (1–5 from ‘very good’ to ‘very poor’): poor (mean score <2.5), medium (2.5≤mean score <3.5) and good (mean score ≥3.5); SE = standard error.

*Principles P18–P21 were rejected by the majority of experts, i.e. their plausibility was below 50%.

Comparing the ratings of evidence and plausibility, we recognised that the existence of supporting evidence was not necessarily a requisite for an ecosystem principle to be plausible. Seven principles obtained intermediate evidence scores and for P16 and P17 evidence was rated as poor. However, this lack of evidence did not translate into a lack of plausibility. It was therefore an advantage to separate the two ratings from each other to distinguish between the experts’ opinions and their evidence-based judgments. However, plausibility was clearly lower overall when evidence ratings were poor (Tables 2 and 3).

Developing principles to link food availability with biodiversity was challenging, and none were rated as plausible (P18 and P20). Experts had strong concerns of oversimplification when it came to the type, quality and amount of organic matter as a source of food and how changes of those parameters affected biodiversity. For biodiversity there was again a concern of over-simplification by omitting information on the spatial scale of biodiversity. In the same way, geographical scale mattered to experts, and lack of information on depth ranges and exact geographical position was criticised. The rating on generality provided a preliminary solution for implementing information on the geographical transferability of principles. Giving experts the chance to express uncertainty about the generality of principles as well as disentangling opinions about the rating of evidence allowed them to express their expectations for submarine canyons based on their research experience, and to transfer widely accepted knowledge from other ecosystems.

### 3.3 Linking principles and services

**Figure 4:**
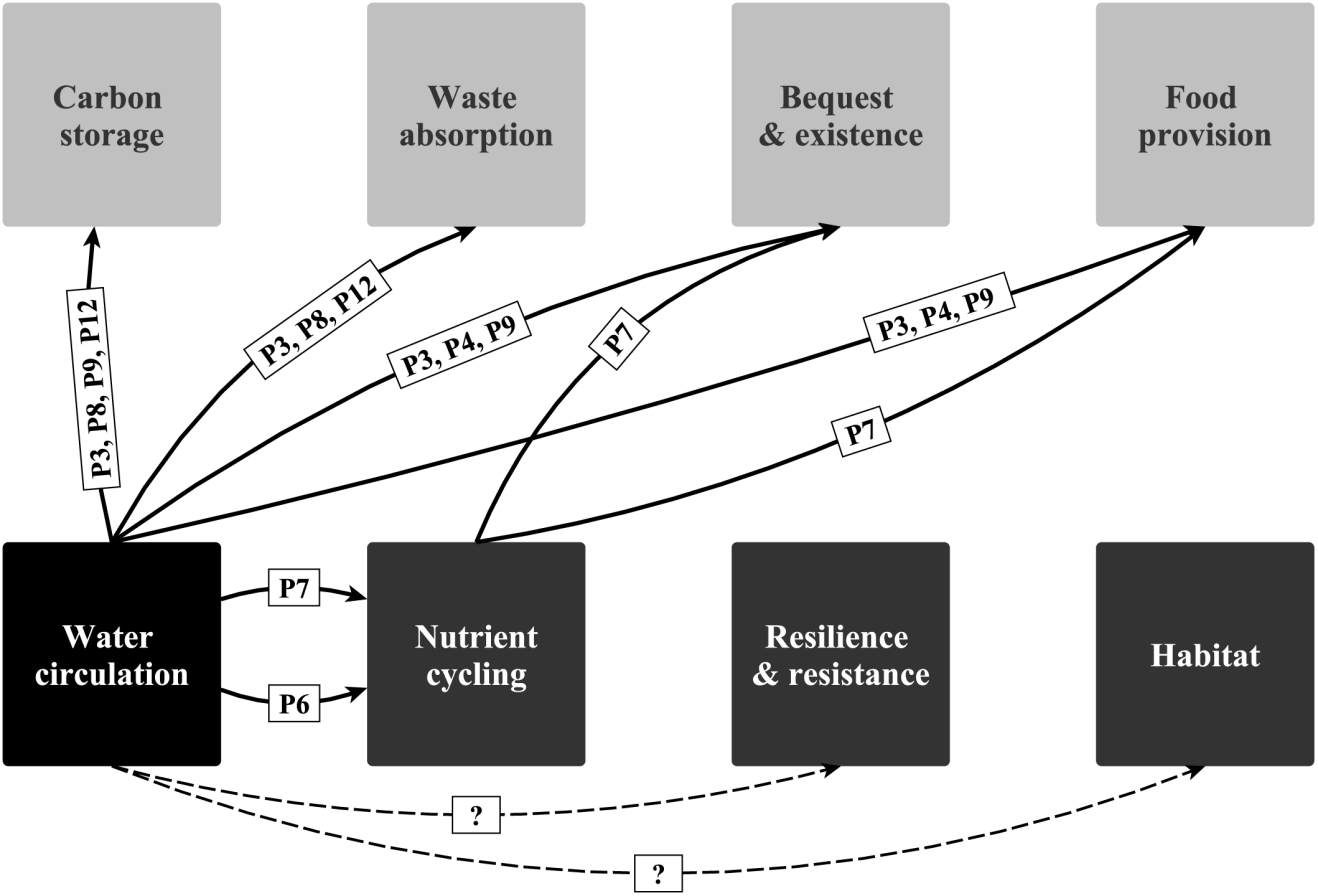
Links between ‘water circulation’ (black box) and other canyon ecosystem services explained through ecosystem principles. The intermediate services are in the lower half (dark grey and black boxes) and final services in the upper half (light grey boxes) of the diagram. Principles are indicated as arrows with their respective ID (cf. explanation in Table S1). Research gaps highlighted as question marks with dotted lines. Principles unrelated to ‘water circulation’ were omitted from this figure.

Many of the principles in Tables 2 and 3 have the capacity to provide information on *where* and *when* principles are likely to operate: for the principles included in Figure 4, particularly P8 and P7 reflect these spatio-temporal components. Other principles such as P3 and P12 explain *how* certain ES are provided and go into more detail on the processes involved. Principles like P17 and P16 that address effects of high biodiversity on ecosystem processes are capable of linking a broader set of ES such as ‘carbon storage’, ‘food provision’, ‘bequest and existence’ and ‘waste absorption’. The ecosystem principles associated with ‘biologically mediated habitat’ were mainly thought to have an effect on biodiversity (e.g. P1 and P13) and to indirectly affect final ES such as ‘food provision’ and ‘bequest and existence values’ (P5).

For sustainable ecosystem management it can be equally important to understand the processes and principles that are involved in the provision of ES, as it is to understand the social and economic benefits of those services. We used ‘water circulation and exchange’ as an example to showcase how ecosystem principles explain links between ecological processes and ES (Figure 4; see also Table S1 for further details). ‘Water circulation and exchange’ has an important connectivity function in the submarine canyon (P11) and upwelling effects can lead to enhanced ‘nutrient cycling’ and as a result enhance productivity (P7 and P6; Figure 4). Further, ‘nutrient cycling’ might be important as an intermediate service for two different final ES, ‘food provision’ and ‘bequest and existence values’, because it can enhance fish abundance (P7). The ‘bequest and existence’ value can arise through the value that people tend to hold for iconic species (including fish), whereas ‘food provision’ relies on the abundance of commercially important fish as a consumptive resource. Trophic relationships, enhanced biomass, maintenance of deep-sea organisms (including non-iconic and non-commercial species) are important processes that sustain ‘bequest and existence’ as well as ‘food provision’ (P3, P4, P9; Figure 4) and should therefore be considered for management purposes. For ‘carbon storage’ and ‘waste absorption’ ecosystem management might be more concerned with other processes such as the transportation of organic and inorganic material, means of transportation, sedimentation rates, storage time, and burial processes that are important in parts of the submarine canyon (P3, P8, P12; Figure 4). How ‘water circulation and exchange’ is linked to ‘resilience and resistance’ as well as ‘biologically mediated habitats’ could not be resolved through the ecosystem principles developed in our workshop. This might be an indication that either too little evidence exists to support any ecosystem principles or that the links with processes that sustain these two ES are too complex to be described in the simplified form of ecosystem principles.

## Discussion

The deep-sea case study for the Nazaré Canyon resulted in new insights on how to address the difficulties of assessing marine ES for ecosystem management purposes, especially when uncertainty is high due to lack of scientific data. In times where the demands on deep-sea resources are increasing, and scientific data on the potential impacts on marine biodiversity is scarce [5], [34]–[36], approaches such as the Ecosystem Principles Approach (EPA) are crucial to draw the link between the ecological and socio-economic dimensions of the ecosystem. Currently this linkage is poorly understood, contributing to the under-valuation of deep-sea ecosystems which is likely to undermine conservation efforts. We briefly outline the utility of expert consultation under these circumstances and reflect on the ability of the EPA to integrate more ecology into the assessment of ES and into the decision-making process. We first discuss how the EPA can help to communicate the overall importance of deep-sea ecosystems for the provision of ecosystem services. Further, we explain how the EPA can improve marine ecosystem-based management by promoting the inclusion of information on ecosystem processes into an ecosystem services assessment.

### 4.1 Communicating ecological values

The EPA has the ability to broaden access to ecological knowledge so that decision makers are not dependent on science advisors alone, but can take informed decisions on the basis of simplified ecological knowledge made available to them [37], [38]. This broadening of access could also assist in increasing information about deep-sea ES amongst the general public. The process of developing ecosystem principles for the submarine canyon environment made clear that experts were able and willing to make predictions on connections between ES, generalisations on important canyon processes, and to link canyon characteristics to effects on ES. Also, while there remain research gaps and uncertainties, the list of ecosystem principles presented in Tables 2 and 3 provides the best available science knowledge to date, presented in easily understandable units of information. The list includes spatial and temporal information, as well as information on how the principles influence the supply of ES. We found that it was very important for the participating scientists to distinguish between their opinions (i.e. plausibility of the principles) and the (less subjective) rating of the existing scientific evidence base to inform principles. It was not imperative for principles to have a good evidence base, but rather to be generally accepted as plausible (cf. P5, P17 and P16). Herein lies a predictive strength of the EPA, in backing up the uncertainty associated with deep-sea science with a consensus-based approach, thereby decreasing uncertainty about ES linkages. The generality of principles was equally important, accounting for concerns that some ecosystem principles were valid on a larger scale than others i.e. ‘general deep-sea principles’ or ‘shelf-incising canyon specific principles’. This additional type of information is crucial to highlight the ability of the EPA to transfer principles to other submarine canyons or even other deep-sea ecosystems. The majority of experts rated the principles as either very broadly applicable to the deep sea or to submarine canyons in general, irrespective of the type of canyon. The broad applicability of principles was thought to be an effect of reducing the complexity of ecological information.

We share the view of one workshop participant who stated that it will be difficult to determine when the list of ecosystem principles is complete. New evidence, the inclusion of researchers with different academic backgrounds, and assigning more time to the task might increase the number of principles on the list. Thus including a broad range of disciplines into the principle development process is crucial. Nonetheless, there exists an asymptotic relationship between effort expended and the number of principles, where spending more time on identifying and reviewing submarine canyon principles might increase the detail of principles, but not their utility for management decisions. The EPA’s utility lies not in providing large amounts of detail, but in providing meaningful, concise information to better understand the overall functioning of the ecosystem in conjunction with the ES it provides. The EPA is based on what we know today and the ecosystem principles in this paper cover a broad range of topics, with further workshops or surveys being likely to provide diminishing returns of new principles to our established list.

There are many aspects of the deep sea that the science community remains uncertain about, but the EPA helped to focus and assimilate known information with the underlying ecosystem processes that are better understood and agreed on. However, the fact that some ecosystem principles were discarded (Table 3) indicated that there remain gaps in understanding on how canyon biodiversity is influenced by current regimes, and by different types and quality of organic material, and also the importance of recruitment processes between deeper and shallower waters. The high specificity and complexity of these and other processes might not allow us to develop these processes as ecosystem principles at the current time.

### 4.2 Utility of the Ecosystem Principles Approach

The deep sea is hard to sample and poorly understood, yet we were able to draw on experts’ knowledge and condense what they know about submarine canyons, one of the deep-sea’s biodiversity hotspots. We showed that the EPA, in combination with a Delphi process, can be a useful tool for working at the fringes of our current knowledge, using collective expert opinions to evaluate and arbitrate on the content of ecological understanding. Through this process we can also highlight where knowledge and hence research gaps lie, and where funding is needed. The EPA might be seen as a balancing act between the precautionary principle on the one side and economic reasoning on the other. The precautionary principle as framed in Rio in 1992 states that lack of scientific certainty shall not be used as an excuse to postpone actions that might prevent environmental degradation [39]. Lubchenco [37] lists guidance for decision-making under uncertainty as one of the roles that science should play in society. This might include reliance on more subjective approaches such as the EPA to support more holistic decision-making in marine resource management until we have a greater body of scientific evidence to prove or disprove what researchers have outlined as ecosystem principles.

The principles for ‘water circulation and exchange’ (Figure 4) demonstrate the EPA’s ability to provide information on the ecological value of the ecosystem, and how these are linked to the kinds of final ES which economists are likely to value in monetary terms. The approach does not focus on final ES alone, but provides information at multiple levels without losing sight of the indirect impacts on ecosystems through intermediate services and underlying processes, and of the multiple connections between ecosystems. By linking processes with services through ecosystem principles, we draw the attention towards the network character of ecosystems. The highly interlinked nature of this network means that it is actually far from straightforward to categorise and separate services for ES valuation, especially given the multitude of connections within the marine environment. This is so irrespective of whether social, ecological, or economic definitions of ‘value’ are assessed. Presenting the information on final and intermediate services together with their underlying processes in a network style can better inform future management scenarios with more realistic ecological information than assessments that are limited to final ES alone.

Current ES valuation frameworks suggest that intermediate services should be valued only in terms of the final services that they support and not be included directly in a valuation of ES flows to avoid double-counting of their social or economic value [25], [40]–[42]. The resulting requirement for effective management is that underlying processes and linkages are sufficiently well understood [23] and the ES they support are provided within the managed area [5]. However, the spatial and temporal distances between marine intermediate and final services can span millennia and act on a global scale, as is the case with the ocean nutrient cycle [35], [43]. Marine ecosystems are highly connected systems with many processes being important for the provision of intermediate and final ES and crossing ecosystem boundaries [5]. Hence, if intermediate and final ES are spatially separated, the chances are that recommendations which focus only on final ES will be poor for marine resource management [22], [44]. The field of ES valuation has its roots in terrestrial ecosystems where ecosystems with their services and processes are less open than in marine ecosystems [45], [46]; valuation approaches might have to be adapted for the marine environment to account for its higher connectivity. Also, to capture the holistic value of intermediate services, we would have to successfully value all final ES (including cultural ES values and other non-marketed ES), which is still more of a research aspiration rather than a currently-achievable outcome. A failure to recognise the contribution of intermediate services for the ES valuation in ecosystems where they dominate, such as the deep sea, will likely lead to misguided policy decisions [47].

The EPA should be seen as an addition to baseline ecological research and economic ES valuation, rather than as a substitute for either. The EPA’s advantage lies where monetary valuation reaches its limits, in highlighting links between ES and their underlying processes, and in linking intermediate services with final ES. While economic ES values can help set marine management priorities that are socially and economically desirable (Daily et al., 2009), the EPA focusses on the ecological ‘value’ of the ecosystem and can provide important information on how such management priorities can be achieved. Where economic values require empirical links to well-being and monetary quantification, ecological ‘value’ is more focussed on the importance of the ecosystem processes and characteristics that lead to such economically valued benefits being produced.

### 4.3 Future research opportunities and lessons learnt

The EPA might not only enhance the availability of ecological information and its uptake by decision makers, but can also improve how research results are shared across disciplines. Inter- as well as trans-disciplinary collaborations are complicated by the existence of language barriers. The use of different key terms or jargon restricts access to the pool of knowledge to only a small number of experts. The lack of frameworks that translate research findings into understandable and meaningful formats has been described as one of the major reasons why information might not reach beyond disciplinary boundaries [48], [49]. Different methodologies, attitudes and perceptions between disciplines might further decrease the flow of scientific evidence [38], [48]. Thus to allow economists, geologists, biologists, oceanographers and other disciplines involved in marine science to share information it would be beneficial to work on a global matrix of ecosystem principles similar to the ES valuation databases provided by the Marine Ecosystem Services Partnership (MESP; URL: www.marineecosystemservices.org; last access August 2013) which gathers studies on monetary ES values. In contrast to the MESP database, the EPA would be able to add to the evidence base not just on economic and social, but also on ecological values. Also, a more extensive dataset on ecosystem principles for marine ecosystems would increase the chances that more complex management scenarios could be developed, such as in Bayesian belief networks (BBNs), which depend heavily on the availability of baseline information on ecosystem processes, even though BBNs are able to deal with knowledge gaps when expert knowledge is available [50]. Other fields that are using approaches like habitat mapping as well as biological value mapping might benefit from the EPA as well, given that spatial ecosystem principles were developed [51].

Using the EPA it should be possible to provide more precise temporal and spatial information on ecosystem principles, and to develop management strategies based on the list of principles and evidence on social and economic values and resulting management priorities. Showing the EPA’s potential to improve people’s understanding of ecosystem functioning was beyond the scope of this study, but future research involving the wider public and decision makers would be beneficial to test the effect of such simplified ecological knowledge on their decisions.

Three insights in particular emerge from our interdisciplinary workshop, which might improve future marine conservation initiatives and their acceptance:

1. To further the field of marine ES valuation it would be beneficial to acknowledge that the traditional approaches to ES valuation, which have their roots in terrestrial research, might not be easily transferable to a highly linked marine environment. Marine ecosystem boundaries are much less clearly defined than in terrestrial environments, and ES flows are less easily traceable. We might therefore need different approaches to valuing ES flows in the marine environment. The EPA is but one potential approach to improve integration of ecological values with social and economic values.
2. The precautionary principle demands that we are cautious with our exploitation of the environment, but in the same time that management recommendations are made on a timely basis to the best of our knowledge, without postponing decisions for indefinite time until more certainty has been gained. The marine science community should more willingly embrace its important societal role in providing recommendations for nature conservation management with the support of social science approaches.
3. We propose greater transparency in decisions on the conservation importance of marine areas. It should be possible to enhance understanding of the social, ecological as well as the economic values of certain areas, and to justify their protection, by providing easy understandable information on marine ES and how they relate to underlying ecosystem processes.

## Supporting Information

Table S1 (DOCX)
