# Supplementary material for: How Can We Identify and Communicate the Ecological Value of Deep-Sea Ecosystem Services?
Source: PLoS One. 2014 Jul 23;9(7):e100646. doi: 10.1371/journal.pone.0100646 (PMC4108315; doi:10.1371/journal.pone.0100646)
Supplement: Table S1 — (DOCX) [file pone.0100646.s001.docx]

**Table S1: Linking ecosystem processes and ecosystem services*.**

| **Ecosystem services link** | **Ecosystem principles involved** | **Underlying ecosystem processes** |
| --- | --- | --- |
| Water circulation  🡪 Carbon storage | **P3** (The strength of large scale transportation events varies and occurrence ranges from a yearly to decadal pattern. They can be triggered by storms, high sediment load in the water column, cooling and increasing salinity of surface waters, or slope failures.)  **P8** (By transporting large amounts of organic material from the shelf into deeper waters, canyons act as temporary stores of sediment and carbon. It can take decades or even centuries until the transported material reaches the abyssal plain, where it is then deposited on geological time scales.)  **P9** (Food quantity and quality tends to be higher within some canyon areas compared to the surrounding slope. This can enhance the biomass of the benthic and pelagic fauna.)  **P12** (Sediment, organic material, and pollutants that are transported alongshore get trapped by the canyon and transported down the canyon slope.) | Connectivity (from A to B)  Means of transport (when & how)  Transport of organic material  Enhanced biomass  Deposition of organic material  Burial by sedimentation/organisms  Storage over time |
| Water circulation  🡪Waste absorption | **P3** (The strength of large scale transportation events varies and occurrence ranges from a yearly to decadal pattern. They can be triggered by storms, high sediment load in the water column, cooling and increasing salinity of surface waters, or slope failures.)  **P8** (By transporting large amounts of organic material from the shelf into deeper waters, canyons act as temporary stores of sediment and carbon. It can take decades or even centuries until the transported material reaches the abyssal plain, where it is then deposited on geological time scales.)  **P12** (Sediment, organic material, and pollutants that are transported alongshore get trapped by the canyon and transported down the canyon slope.) | Connectivity (from A to B)  Means of transport (when & how)  Deposition of (organic) pollutants  Deposition of waste material  Burial by sedimentation/organisms  Storage over time |
| Water circulation  🡪Bequest & existence | **P3** (The strength of large scale transportation events varies and occurrence ranges from a yearly to decadal pattern. They can be triggered by storms, high sediment load in the water column, cooling and increasing salinity of surface waters, or slope failures.)  **P4** (The transport of organic material from shallower waters to the deep seabed, which is mainly driven by large scale transportation events, is an important source of food for deep-sea organisms.)  **P9** (Food quantity and quality tends to be higher within some canyon areas compared to the surrounding slope. This can enhance the biomass of the benthic and pelagic fauna.) | Connectivity (from A to B)  Means of transport (when & how)  Maintenance of organisms  Trophic relationships  Enhanced biomass (including iconic fauna) |
| Water circulation  🡪Food provision | **P3** (The strength of large scale transportation events varies and occurrence ranges from a yearly to decadal pattern. They can be triggered by storms, high sediment load in the water column, cooling and increasing salinity of surface waters, or slope failures.)  **P4** (The transport of organic material from shallower waters to the deep seabed, which is mainly driven by large scale transportation events, is an important source of food for deep-sea organisms.)  **P9** (Food quantity and quality tends to be higher within some canyon areas compared to the surrounding slope. This can enhance the biomass of the benthic and pelagic fauna.) | Connectivity (from A to B)  Means of transport (when & how)  Maintenance of organisms  Trophic relationships  Enhanced biomass (including commercially important species) |
| Water circulation  🡪Nutrient cycling | **P7** (The canyon topography affects up- and down-welling of water masses at the continental margin. Upwelling events around the canyon head enhances productivity locally; as a result fish abundance can be higher.)  **P6** (Canyons can enhance the mixing of water masses and as a result influence the exchange of nutrients, heat and salt between the shelf and the deep sea.) | Connectivity (from A to B)  Upwelling effects  Enhanced productivity |
| Water circulation  🡪 Nutrient cycling  🡪Bequest & existence | **P7** (The canyon topography affects up- and down-welling of water masses at the continental margin. Upwelling events around the canyon head enhances productivity locally; as a result fish abundance can be higher.) | Enhanced fish abundance (including iconic species) |
| Water circulation  🡪Nutrient cycling  🡪Food provision | **P7** (The canyon topography affects up- and down-welling of water masses at the continental margin. Upwelling events around the canyon head enhances productivity locally; as a result fish abundance can be higher.) | Enhanced fish abundance (including commercially important species) |

* All ecosystem principles involved in linking ‘water circulation’ with other services are explained as supplementary information to Figure 4.
